# Quantitative temporal analysis of posterior oral spillage in a dual-task for individuals with Parkinson’s disease

**DOI:** 10.1590/2317-1782/e20250119en

**Published:** 2026-01-26

**Authors:** Laura Mochiatti Guijo, Rarissa Rúbia Dallaqua Felix, Paula Cristina Cola, Roberta Gonçalves da Silva, Suely Mayumi Motonaga Onofri

**Affiliations:** 1 Laboratório de Disfagia, Departamento de Fonoaudiologia, Universidade Estadual Paulista – UNESP - Marília (SP), Brasil.

**Keywords:** Deglutition, Deglutition Disorder, Parkinson Disease, Dual-Task, Endoscopy

## Abstract

**Purpose:**

To compare the POS time in individuals diagnosed with PD in the conditions of isolated deglutition (ID) and dual-task deglutition (DD) for different consistencies and volumes.

**Methods:**

A total of 576 swallows edited from fiberoptic endoscopic evaluation of swallowing (FEES) of 16 individuals, both sexes, at different PD stages based on the Hoehn & Yahr (H&Y) modified scale, aged 64 to 85 years (mean ± standard deviation: 72.4 ± 6). They underwent FEES with isolated deglutition (ID) and dual-task deglutition (DD) to analyze the POS time in swallowing. An otorhinolaryngologist performed the FEES, offering standardized consistencies at levels 0 – thin; 2 – mildly thick; and 4 – extremely thick, based on the International Dysphagia Diet Standardization Initiative (IDDSI). All food consistencies were dyed with blue artificial food coloring and offered 5 mL and 10 mL in disposable spoons. After adequate training, the quantitative temporal POS analysis for both deglutition conditions was performed using specific software. Data was analyzed through the Statistical Package for the Social Sciences (SPSS) with a significance level of 0.05 (5%). The Mann-Whitney test compared the ID and DD POS time.

**Results:**

The POS time was statistically significantly different for 5 mL of consistency level 4 (ID = 912 ms and DD = 2.044 ms) (p-value = 0.007).

**Conclusion:**

The results indicated that there was significant difference in the POS time between ID and DD only at 5 mL of consistency level 4 for individuals with PD at performing the cognitive-motor dual-task proposed.

## INTRODUCTION

Parkinson’s disease (PD) can cause swallowing difficulties^(1,2)^, and its efficiency and safety require attentional resources shared with cognitive and motor behaviors^(3)^ in family meals^(4)^, when they handle cutlery and view digital media^(5-7)^.

A phenomenon known as dual-task interference may explain how two tasks performed simultaneously may deteriorate the performance in one or both tasks, including deglutition^(8-10)^. Recently published study evidenced that individuals with PD performed simultaneous tasks that require divided attention, with reduced swallowing efficiency due to the longer oral phase^(11)^. Even though the mentioned study^(11)^ being innovative in the area of oropharyngeal dysphagia due to the application of a dual-task paradigm, aspects such as a small sample of only 10 individuals with PD, at different stages of the disease, the lack of a control group and the results without significant differences regarding the response time and duration of the anticipatory and oropharyngeal phases of swallowing are important limitations that demonstrate the need for future studies, with more refined designs. Thus, more precise results may be found to prove the effect of secondary tasks performed during swallowing.

The influence of the dual task on swallowing of individuals with PD has been analyzed through instrumental swallowing examinations, such as videofluoroscopic swallowing study (VFSS)^(12)^ and fiberoptic endoscopic evaluation of swallowing (FEES)^(3,5,13)^. One of the parameters they analyze is the presence of posterior oral spillage (POS), associated with the loss of oral control of the food bolus^(14)^. As the oral phase of swallowing requires the cortical processing of cognitive demand, PD patients’ cognitive impairments may result in impaired of this phase of swallowing.

The cognitive function is predominantly performed by the frontal cortex, which is involved in the voluntary phase of swallowing. Findings regarding the effects of the dual-task may be clinically relevant to demonstrate changes in the efficiency and/or safety of the swallowing during instrumental swallowing examinations, improve the quality of life of dysphagic individuals regarding that meals occur in a social context^(15)^, and elucidate contributions to the therapeutic planning and guidance for caregivers and family members aiming at continuity of speech therapy objectives and care of the patients. Thus, this study aimed to compare the POS time in individuals diagnosed with PD in the conditions of isolated deglutition (ID) and dual-task deglutition (DD) for different consistencies and volumes.

## METHOD

This is a clinical, cross-sectional, observational, inferential study conducted at ^1^Dysphagia Lab – Speech, Language and Hearing Sciences Department, São Paulo State University – UNESP-Campus of Marília/SP-Brazil and was approved by the Ethics Committee under no. 5.166.265. All participants signed an informed consent form.

### Participants

The study included 16 individuals aged 64 to 85 years (mean ± standard deviation: 72.4 ± 6) of both sexes, whose PD diagnosis was confirmed by clinical neurological evaluation at the Rehabilitation Center. The mean time of the PD’s diagnosis of the individuals was 4.75 years. All participants were evaluated at the ON phase of medication, that is, regarding the time window (1-2 hours after intake) in which medication adjusted the dopamine level in the nervous system and reduced the effects of motor symptoms, such as slow movements, joint stiffness, resting tremor, and non-motor symptoms such as pain, fatigue, anxiety and depression.

The inclusion criteria were: PD diagnosis conducted by a neurologist, no other associated neurological diseases; age from 60, considering the classification of older individuals^(16)^; no swallowing problems or speech-language-hearing diagnosis of oropharyngeal dysphagia; up to stage III on the modified Hoehn & Yahr (H&Y) scale^(17)^ or more severe PD stages but able to perform the swallowing dual task; no laryngeal or esophageal malformation or surgical intervention; ability to respond to the evaluator's commands to perform the dual task during the instrumental swallowing examination.

Exclusion criteria were: use of tracheostomy, clinical instability, evident structural changes that compromise swallowing visualization, history of head, and neck structural damage, other neurological disorders associated.

An otolaryngologist performed the FEES and the laryngeal sensitivity was tested by touching the distal tip of the endoscope to the arytenoids and bilateral aryepiglottic folds, as proposed by previous study^(18)^ and the sensitivity was classified as bilateral presence, unilateral presence, or bilateral absence. A sensitivity analysis was performed after the exam by two evaluators: the otolaryngologist that performed the exam and a speech language therapist with several years of experience in the area of dysphagia.

The participants' characterization regarding age, sex, PD stage (based on the modified H&Y), and laryngeal sensitivity is shown in Chart 1.

**Chart 1:** Participants' characterization regarding age, sex, diagnosis time of Parkinson's disease, stage of Parkinson's disease, and laryngeal sensitivity

**Individuals with PD**	**Age**	**Sex**	**Diagnosis time of Parkinson's disease (years)**	**Stage of PD**	**Laryngeal sensitivity**
1	81	M	5	5	Present bilaterally
2	65	F	2	1	Present bilaterally
3	71	F	4	1.5	Present bilaterally
4	79	M	4	1	Present bilaterally
5	78	F	4	2	Present bilaterally
6	84	F	5	1.5	Present bilaterally
7	77	M	5	2.5	Present bilaterally
8	70	M	5	2	Present bilaterally
9	66	F	4	2.5	Present bilaterally
10	68	M	7	3	Present bilaterally
11	64	M	7	1.5	Present bilaterally
12	76	M	6	2.5	Present bilaterally
13	68	F	5	1.5	Present bilaterally
14	69	F	2	1	Present bilaterally
15	73	F	5	2	Present bilaterally
16	70	M	6	1.5	Present bilaterally

**Caption:** PD = Parkinson's disease; M = Male; F = Female

### Procedures

#### FEES – Isolated deglutition (ID) and dual-task deglutition (DD)

The otolaryngologist performed the FEES with a Pentax® nasofibroscope, coupled to the Pentax® micro camera system and the Pentax® light source, model LH-150 PC, capturing images with the Zscan 6.0 software. Participants were instructed to remain seated, and the endoscopy was performed through the most patent nasal cavity, without topical anesthetic to avoid changes in local sensitivity. Laryngeal sensitivity was confirmed by touching the distal end of the endoscopy to the bilateral aryepiglottic folds/arytenoids and observing the vocal fold adduction reflex.

Food consistencies for the FEES were standardized and equated to terminology by levels 2 – mildly thick, 4 – extremely thick, and 0 – thin, based on the International Dysphagia Diet Standardization Initiative (IDDSI)^(19)^. Levels 4 and 2 consistencies were prepared with liquid and a peach-flavored diet juice by adding an instant food thickener with modified cornstarch and maltodextrin. Level 0 consistency was water at room temperature. All food consistencies were dyed with blue artificial food coloring and offered 5 mL and 10 mL in disposable spoons.

The cognitive-motor dual-task in this study was the random command to elevate the right/left upper limbs during swallowing, similar to the movement of bringing a spoon toward the oral cavity in an autonomous feeding situation; the command could be repeated, even asking to elevate the same limb. The evaluator offered food on a spoon; regarding the temporal relationship between the verbal command and swallowing, after participants captured the food in their mouths, they were instructed to swallow it and raise the arm as requested, immediately after the verbal command and, after each completion of the requested limb elevation movement, the dual-task was started again.

Regarding the randomness of the verbal commands for elevating the right/left upper limbs, these were distributed into 12 lists manually created. It was not required or considered that the individuals correctly identified the upper limb to be elevated in all verbal commands, but they were advised to pay attention to the commands for the best possible execution.

### FEES analysis

Altogether, 576 swallows were edited from the FEES for both conditions (ID/DD), considering three swallows for each food consistency and volume. The distribution of the total number of ID and DD swallows per food consistency and volume is described below.

#### Quantitative temporal analysis of the POS

The quantitative temporal analysis of the POS in ID/DD was performed using specific software, which recorded the POS time in milliseconds through the analysis of video frames and serialized swallows^(20)^. The quantitative temporal analysis was trained mainly with appropriate software use and delimitation of anatomical points and the presence/start/end of the POS in FEES^(20)^. The quantitative temporal analysis of the POS for this study was performed by only one judge, after adequate training, as both the software used and the method of measuring swallowing parameters demonstrated excellent agreement between senior and junior evaluators, based on a previous publication^(21)^.

In the training, the lead researcher initially analyzed the FEES in the software, verifying the POS time in approximately five edited FEES, considering each consistency and volume offered to individuals with PD. The software operation and parameter analysis were discussed for 12 hours. They performed frame-by-frame analysis with the FEES edited, digitized, and analyzed in milliseconds with an acquisition rate of 29.97 frames per second, determining the POS start when the food bolus reached the vallecula and its end when the start of the white-out (WO) could be seen^(22)^.

### Statistical analysis

The statistical descriptive and inferential analyses used categorized data through the Statistical Package for the Social Sciences (SPSS). Nonparametric statistical tests were applied due to the lack of guaranteed normal distribution of the main outcome quantitative variables, verified by the Kolmogorov-Smirnov test (N < 100). The Mann-Whitney test compared the POS time in ID and DD with the different food consistencies and volumes. The significance level was set at 0.05 (5%).

## RESULTS

Results regarding the effect of the DD on the quantitative temporal analysis of the POS are shown in Tables 1, 2, and 3 with different consistencies and volumes.

**Table 1:** Analysis of posterior oral spillage time under deglutition conditions for extremely thick (level 4/IDDSI)

DC	Vol	n	Mean (ms) (SD)	Median (Q_1_ - Q_3_)	IQR	CI 95%	p-value
ID	5mL	33	912 (1,082)	434 (100 -1,501)	1,401	543 – 1,281	0.007*
DD		35	2,044 (2,829)	1,201 (634 – 2,236)	1,602	1,107 – 2,981	
ID	10mL	34	1,562 (1,758)	900 (400 -1,802)	1,402	971 – 2,153	0.592
DD		38	1,598 (1,703)	1,185 (559 – 1,602)	1,043	1,057 – 2,139	

Mann-Whitney test compared the POS time in ID and DD for IDDSI level 4, 5 and 10ml: *p < 0.05

**Caption:** DC = deglutition conditions; ID = isolated deglutition; DD = dual-task deglutition; ms = milliseconds; SD = standard deviation; n = number of swallows; mL = milliliter; Q_1_ = quartile 1; Q_3_ = quartile 3; IQR = interquartile range; CI = confidence interval; IDDSI = International Dysphagia Diet Standardization Initiative

**Table 2:** Analysis of posterior oral spillage time under deglutition conditions for mildly thick (level 2/IDDSI)

DC	Vol	n	Mean (ms) (SD)	Median (Q_1_ - Q_3_)	IQR	CI 95%	p-value
ID	5 mL	34	1,135 (1,708)	667 (234 - 993)	759	561 – 1,709	0.077
DD		31	1,580 (2,132)	1,034 (584 – 1,585)	1,001	830 – 2,330	
ID	10 mL	25	1,630 (1,861)	1,468 (267 – 2,035)	1,769	901 – 2,359	0.360
DD		35	1,944 (1,937)	1,068 (534 – 2,469)	1,935	1,302 – 2,586	

Mann-Whitney test compared the POS time in ID and DD for IDDSI level 2, 5 and 10ml: p < 0.05

**Caption:** DC = deglutition conditions; ID = isolated deglutition; DD = dual-task deglutition; ms = milliseconds; SD = standard deviation; n = number of swallows; mL = milliliter; Q_1_ = quartile 1; Q_3_ = quartile 3; IQR = interquartile range; CI = confidence interval; IDDSI = International Dysphagia Diet Standardization Initiative

**Table 3:** Analysis of posterior oral spillage time under deglutition conditions for thin (level 0/IDDSI)

DC	Vol	n	Mean (ms) (SD)	Median (Q_1_ - Q_3_)	IQR	CI 95%	p-value
ID	5 ml	33	861 (965)	567 (200 - 868)	667	532 – 1,190	0.610
DD		30	1,447 (2,48)	434 (235 – 1,126)	891	392 – 2,502	
ID	10 mL	32	1,118 (1,343)	484 (317 – 1,143)	826	653 – 1,583	0.708
DD		36	1,164 (1,509)	534 (225 – 1,326)	1,101	671 – 1,657	

Mann-Whitney test compared the POS time in ID and DD for IDDSI level 0, 5 and 10ml: p < 0.05

**Caption:** DC = deglutition conditions; ID = isolated deglutition; DD = dual-task deglutition; ms = milliseconds; SD = standard deviation; n = number of swallows; mL = milliliter; Q_1_ = quartile 1; Q_3_ = quartile 3; IQR = interquartile range; CI = confidence interval; IDDSI = International Dysphagia Diet Standardization Initiative

After comparing the quantitative temporal analysis of the POS in the ID and DD conditions, it was observed that the POS time was longer for the DD condition, regardless of the food consistencies and volumes.

There was a statistically significant difference only for the 5 mL of consistency level 4, with the mean of the ID condition equivalent to 912ms and the mean of the DD condition equivalent to 2.044ms (p-value = 0.007), as shown in Table 1.

There was no statistically significant difference for consistency level 2, in both food volumes, as shown in Table 2.

There was no statistically significant difference for consistency level 0, in both food volumes, as shown in Table 3.

## DISCUSSION

Published studies on the dual-task interference with the swallowing of PD patients lack a consensus on which dual tasks may interfere with the efficiency and safety of swallowing – i.e., the presence of laryngeal penetration and laryngotracheal aspiration^(3,13)^. These studies used different protocols to offer food consistencies and volumes, making it difficult to agree on the findings on the dual-task interference with swallowing efficiency^(3,11)^ and safety^(5,12,13)^.

Some deglutition studies in the current literature address divided attention in cognitive and motor tasks, with populations with no swallowing changes^(14,19,23)^ and diagnosed with PD^(12,13)^. Studies have found that divided attention due to cognitive and motor tasks affects the swallowing of individuals with PD^(12,13)^. However, the dual-task influence on their deglutition performance has not been widely explored, described, or confirmed, mainly due to questions regarding the type of dual-task that most distract deglutition, the lack of standardized consistencies and volumes to refine the findings, and the lack of randomized deglutition conditions in data collection to avoid biases^(12-14)^. This study aimed to verify whether a dual task affects the POS time in the deglutition of individuals with PD. The initial hypothesis was that individuals with PD would increase the POS time in the DD and perform best in the ID because no motor command would overload the latter.

To date, no studies have been found that verified the effect of dual tasks on POS findings in ID and DD for individuals with or without neurological changes through FEES or VFSS. The studies currently published verified the effects of dual tasks on the swallowing of individuals with PD through FEES, analyzing pharyngeal residues in isolation^(13)^ or all parameters such as POS, pharyngeal residues, penetration, and aspiration through qualitative scales to classify the level of the examination findings^(3)^. However, temporal findings are still scarce in the literature^(24)^.

The results published to date show that dual tasks have not affected swallowing safety^(3,13)^. However, it is important to highlight that the results of this study must consider the outcome used to measure the objective. Studies on dual-task interference have not found impacts on swallowing safety mostly because the presence of penetration and aspiration depends on other biomechanical issues. Thus, the question arises as to whether dual tasks would impact swallowing safety when attention is divided. This study analyzed only the POS in people with PD because it occurs in a voluntary phase of swallowing due to the deficit in oral control^(25-27)^, controlled by cognitive resources and a complex cortical modulation also responsible for performing various dual motor tasks, characterizing it as the best outcome for this type of study.

The POS was present in more than half (over 60%) of the swallows analyzed in this study, regardless of consistency and volume, with a slightly greater occurrence in the DD (over 70% of swallows) with thick consistencies (levels 4 and 2). Although POS may contribute to the occurrence of laryngeal penetration and laryngotracheal aspiration, depending on the degree of oral incoordination and pharyngeal response time, the literature shows that this parameter is a frequent finding in deglutition biomechanics without changes^(28,29)^.

A study investigated through FEES the frequency of POS, pharyngeal residues, laryngeal penetration, and laryngotracheal aspiration in 40 healthy adults (mean age of 38 years) without any diagnosis of neurological or deglutition disorders, offering them soft solid food and thin liquids. It evaluated 967 swallows (479 of soft solid food and 488 of thin liquid), observing the POS in 64% of swallows (65% of solid food and 64% of thin liquid). Thus, the POS normal variation is commonly found in healthy adults during meals^(28)^.

Another study investigated the degree of deglutition impairment by comparing clinical and instrumental evaluations through FEES in 37 healthy older adults aged 60 to 82 years, offering them liquid, pureed, and solid food. The results show a higher occurrence of moderate deglutition impairment, followed by functional swallowing in the clinical evaluation^(29)^. On the other hand, the FEES showed mild and moderate deglutition impairment. The POS in the older population was one of the FEES findings, mainly for solid (70.27%) and pureed (59.46%) food, while the liquid had the lowest POS occurrence (27.03%)^(29)^.

It is noteworthy that the POS for the population studied by Salgado and collaborators is similar to the age range of the individuals in the present study, whose FEES findings may be related not only to the pathophysiology of PD but also to the consequences of aging on deglutition.

Regarding the effect of dual-task interference on POS time in the ID and DD, POS time was longer in the DD, regardless of food consistency and volume. Therefore, the cognitive-motor task interfered with this aspect of deglutition due to the external cognitive demand^(13)^. These findings are consistent with those in the published literature regarding cognitive overload when performing concomitant motor acts, indicating that motor tasks, such as walking, may be affected by external cognitive competition or motor tasks^(30,31)^.

Another aspect currently evidenced by the literature that cannot be disregarded is the fact that the study participants comprised older adults diagnosed with PD. Therefore, the participants in this study have both PD and the physiological swallowing deterioration that impairs the performance of the oral phase of swallowing, with reduced tongue movements (needed for oral propulsion) and delayed pharyngeal response – which can increase the POS to swallow thick consistencies^(32,33)^.

The comparison of the ID and DD POS time in the cognitive-motor dual task with different food consistencies and volumes in individuals with PD found a statistically significant difference only for 5 mL of consistency level 4, with a mean of 912 ms in ID and 2.044 ms in DD. There was a trend towards a significance for consistency level 2, volume of 5 mL, with a mean of 1.135 ms in ID and 1.580 in DD. These comparisons also showed that the POS time was longer in the DD condition for all consistencies, even though there are only significant differences for 5 mL of consistency level 4. Despite the emphasis on the increased POS time in the DD condition for all food consistencies and volumes offered to individuals with PD in this study, there was no comparative analysis of the POS time between consistencies/volumes regarding the same deglutition condition, and this aspect may be analyzed in future studies.

In addition, other limitations stand out in this study: the sample with few participants and the non-randomized deglutition conditions during the FEES examination. These aspects may have influenced the results due to the possible learning effect during the different food offers, causing less interference of the dual task in the POS findings. Future studies may exclude the learning effect by randomizing the deglutition conditions during the FEES. Another limitation was the lack of control regarding the time when they took the medication that helped with the motor symptoms of PD.

The findings of this study may have been more accurate if the participants were in more advanced PD stages. Another important limitation is the failure to perform a clinical evaluation of swallowing, whose findings lead to conclusions about the impact of possible oral phase changes on the pharyngeal phase of swallowing, whereas FEES enables the analysis of pharyngeal findings. Another possibility is to design future studies using the VFSS instrumental examination to visualize food in the oral phase of swallowing.

Further studies are doubtlessly needed to investigate the clinical implications of deteriorated oropharyngeal deglutition due to dual tasks. However, published studies on the interference of simultaneous motor actions play a crucial role since individuals with PD have great difficulty with automatic movements from the early stages of the disease, and these difficulties worsen when combined with other motor tasks^(13)^. These observations suggest that normal movement patterns are not lost but are interrupted by concurrent motor and cognitive tasks^(33)^.

Future research is needed to answer several questions regarding the effect of dual tasks on efficient and safe swallowing, especially in individuals with reduced neuroplasticity as occurs with the advancement of PD stages. However, speech-language-hearing guidance and management to reduce distractions during meals and direct attention to swallowing cannot be disregarded. This study also elucidates the need to include a dual-task protocol in instrumental swallowing examinations, especially for populations with cognitive decline, considering the limitation of cognitive resources when performing concomitant tasks. Finally, dual-task situations could also be applied early in behavioral swallowing therapy to maintain a functional reserve during meals.

## CONCLUSION

The results indicated that there was significant difference in the POS time between ID and DD only at 5 mL of consistency level 4 for individuals with PD at performing the cognitive-motor dual-task proposed in this study.
